# What is next after the genes for autoimmunity?

**DOI:** 10.1186/1741-7015-11-197

**Published:** 2013-09-04

**Authors:** John Castiblanco, Mauricio Arcos-Burgos, Juan-Manuel Anaya

**Affiliations:** 1 Center for Autoimmune Diseases Research (CREA), School of Medicine and Health Sciences, Universidad del Rosario, Carrera 24 #63-C-69, Bogota, Colombia; 2 Doctoral Program in Biomedical Sciences, Universidad del Rosario, Bogotá, Colombia; 3 Center for Personalized Medicine, South Texas Veterans Health Care System, University of Texas Health Science Center at San Antonio, San Antonio, TX, USA; 4 Department of Medicine, University of Texas Health Science Center at San Antonio, San Antonio, TX, USA; 5 Genome Biology Department, John Curtin School of Medical Research, ANU College of Medicine, Biology and Environment, The Australian National University, Canberra, Australia

**Keywords:** Autoimmunity, Common, Genetics, Genomics, Personalized, Predictive medicine, Polyautoimmunity, Translational medicine

## Abstract

Clinical pathologies draw us to envisage disease as either an independent entity or a diverse set of traits governed by common physiopathological mechanisms, prompted by environmental assaults throughout life. Autoimmune diseases are not an exception, given they represent a diverse collection of diseases in terms of their demographic profile and primary clinical manifestations. Although they are pleiotropic outcomes of non-specific disease genes underlying similar immunogenetic mechanisms, research generally focuses on a single disease. Drastic technologic advances are leading research to organize clinical genomic multidisciplinary approaches to decipher the nature of human biological systems. Once the currently costly omic-based technologies become universally accessible, the way will be paved for a cleaner picture to risk quantification, prevention, prognosis and diagnosis, allowing us to clearly define better phenotypes always ensuring the integrity of the individuals studied. However, making accurate predictions for most autoimmune diseases is an ambitious challenge, since the understanding of these pathologies is far from complete. Herein, some pitfalls and challenges of the genetics of autoimmune diseases are reviewed, and an approximation to the future of research in this field is presented.

## Introduction

The everlasting vision of a predictive and preventive framework for disease assessment has pushed the medical sciences to search for new means to manage health care and translate basic research into clinical practice. However, as we dig deeper into the cell and disease mechanisms, the path is not always clear because each new achievement and tool leads to more intricate definitions and targets [1]. Likewise, the cost and configuration of health care plans do not take into consideration the move towards personalized medicine, due in part to the lack of interaction between basic and clinical research. Advances in technology are now prompting this interaction, preparing for more realistic bench to bedside implementation [1-3].

The lack of pathognomonic diagnostic tools and clear-cut diagnostic criteria for complex conditions exposes patients to a bureaucratic limbo, stuck in the system in search of an accurate and complete diagnosis to receive appropriate treatment. Clinical pathologies lead us to consider disease as either an independent entity or a diverse set of traits governed by common physiopathological mechanisms that are prompted by environmental assaults throughout life [4,5]. Autoimmune diseases (ADs) are not an exception. Though the damage to tissues and organs arising from the loss of tolerance is the common attractor to ADs, they represent a diverse collection of diseases defined by their demographic and epidemiological profile, genetic configuration of susceptibility, environmental spectrum and clinical manifestations [4]. Although research more often focuses on a single disease (phenotype), autoimmune phenotypes could represent heterogeneous outcomes of genes underlying similar immunogenic mechanisms, by either cross-phenotype association or by pleiotropy [4,6]. In this sense, clinical observations indicate the possible shift from one disease to another, or the fact that more than one AD may coexist in a single patient (that is, polyautoimmunity) or in the same family (that is, familial autoimmunity) [7].

This article provides a glimpse of the current and future directions for autoimmunity and ADs, discussing the many variables affecting the potential use and application of genetic, evolutionary, demographic, environmental and immunopathological information that could be used for prediction, prevention and eventually treatment of ADs.

### The genetic component of ADs

**Figure 1:**
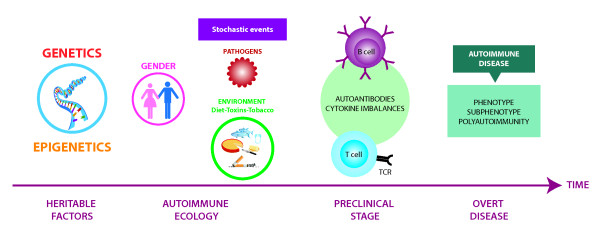
**Etiopathogenesis of autoimmune diseases.** Outline showing the plausible stages for a multifactorial etiology to develop over time. Each stage shows the known phenomena that cumulatively will be the causative scenario for the onset of disease(s). First, heritable factors (that is, genetics, including ancestry, and epigenetics) impact over the life of the individuals. They converge and interact to create and increase or decrease the liability an individual would have to develop the phenotype depending on risk and protective effects. Women are more affected than men. Second, the autoimmune ecology is characterized by the interactions between an individual and its environment, which acting stochastically will also influence the risk and course of disease. The additive effects of heritable and environmental risk factors favor the loss of autoimmune tolerance. Then, a preclinical stage characterized by B and T cell dysregulation arises. This third phase may take years before the phenotype becomes clinically evident. Adapted from Anaya [4] (with permission from Elsevier). This model may apply to all complex diseases. TCR, T cell receptor.

As multifactorial conditions, ADs develop from the cumulative effect of diverse events on the immune system. It is now clear they do not begin at the time of clinical appearance but rather many years before (Figure 1). This window of clinical silence offers the possibility of predicting ADs [8].

**Figure 2:**
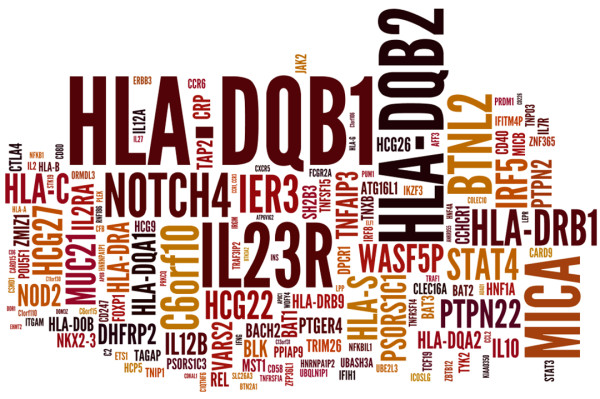
**Weighted list created from the reported significant mapped genes in the current genome-wide association studies curated from the National Human Genome Research Institute and the database of genotypes and phenotypes.** The word cloud shows the frequency of genes and its associated variants relative to their font size using a freely available java applet [24]. Both databases (accessed April 2013) [25,26] were queried taking into account *P-values* reported for the genetic variants associated with autoimmune disease. For the National Human Genome Research Institute, a total of 12,064 genetic variants were encountered, out of which 1,370 were variants significantly associated with autoimmune disease susceptibility. In the database of genotypes and phenotypes, out of 31,246 reported variants, 972 were mutually exclusive from the National Human Genome Research Institute, for a grand total of 2,342 genetic variants related to genes associated in a genome-wide association study of any population. The autoimmune diseases of interest were autoimmune thyroid disease, Behcet’s disease, celiac disease, rheumatoid arthritis, inflammatory bowel disease, juvenile rheumatoid arthritis, Kawasaki disease, multiple sclerosis, primary biliary cirrhosis, primary sclerosing cholangitis, psoriasis, systemic sclerosis, systemic lupus erythematosus, type 1 diabetes and vitiligo.

Familial aggregation is observed in ADs, but the prevalence in close relatives of affected individuals is usually lower than would be expected if these conditions were Mendelian-like [9]. Recurrent associations have been reported in the literature [10-12]. The diseases of this aggregated pattern share similar genetic risk factors, including the major histocompatibility complex and also non-major histocompatibility complex variants [13-15] (Figure 2). A higher concordance rate of ADs in monozygotic than in dizygotic twins supports a significant effect of genes additively contributing to autoimmunity [16]. Although there is higher concordance in monozygotic twins, environment, stochastic phenomena and exposure still result in discordance in disease thresholds among such twin pairs [17]. Reported heritability, based on available twin concordance rates and prevalence estimated for ADs as a group, ranges from 0.008 for systemic sclerosis to 1.0 for Crohn’s disease, with a median value close to 0.6 [18]. ADs are not inherited in a classical Mendelian pattern, but instead have a complex, yet incompletely defined mode of inheritance [19-21]. Further study is needed on environmental and epigenetic factors to clarify their role and effect to allow a greater understanding of their influence, along with genetics, in defining the onset and progression of ADs. The National Institute of Environmental Health Sciences through expert panel workshops has started revisions of such factors to support this growing field of autoimmunity research [22]. For instance, exposure to organic solvents has been shown to affect the risk to develop ADs [23].

Age remains an important topic in autoimmunity, not only because of the biological implications of aging on the immune system but also because of the setback it constitutes for epidemiologic studies [27]. Further complications arise when two diseases are so far apart at their time of diagnosis that a rigorous follow-up becomes imperative to find co-occurrence in one patient [28].

The reason for a major prevalence of ADs among women is poorly understood. The more frequent the AD and the later it appears, the more women are affected [29]. The most convincing explanation of female-biased autoimmunity remains the hormonal theory. Hormones such as estrogens and prolactin have been studied for increasing susceptibility to ADs and can affect both innate and adaptive immune systems [29]. Generally, women have a stronger humoral and cellular immune response than men.

In complex traits, allelic architecture challenges the identification of common and rare genomic variants and their potential effect on risk or protection to develop ADs [15]. Several strategies have been considered to dissect variants either associated or co-segregating with ADs (that is, association or linkage approaches such as family-based co-segregation analysis) [9,15]. For association studies, two approaches are available: genome-wide association studies (GWAS) and candidate gene studies. The genome-wide association approach is usually hypothesis-free whereas the candidate gene is hypothesis-driven.

**Figure 3:**
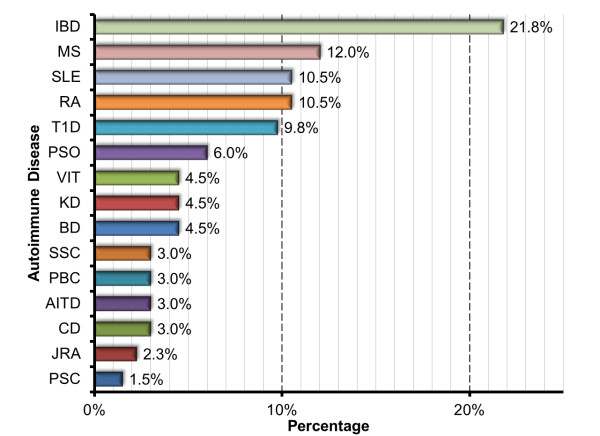
**Histogram showing the percentage of autoimmune diseases with significant reported genetic variants in the current genome-wide association studies curated from the National Human Genome Research Institute and the database of genotypes and phenotypes.** Both databases [25,26] were accessed in April 2013. AITD, autoimmune thyroid disease; BD, Behcet’s disease; CD, celiac disease; IBD, inflammatory bowel disease; JRA, juvenile rheumatoid arthritis; KD, Kawasaki disease; MS, multiple sclerosis; PBC, primary biliary cirrhosis; PSC, primary sclerosing cholangitis; PSO, psoriasis; RA, rheumatoid arthritis; SCL, systemic sclerosis; SLE, systemic lupus erythematosus; SSC, systemic sclerosis; T1D, type 1 diabetes; VIT, vitiligo.

A leap forward towards the recognition of more genes coincided with the advent of high-throughput genotyping technologies and genetic variation repositories, which allowed the use of large sample cohorts to screen for new variants. GWAS interrogate the vast majority of known common polymorphisms [30,31]. This strategy led to a broad array of studies of different AD cohorts (Figure 3), aiming to disclose either new genes or loci associated with ADs or to replicate previously reported associations (Figure 2). Guidelines for the design, quality control and interpretation of GWAS have been presented elsewhere [32-34], as well as novel approaches to study shared genetic factors (for example, cross-phenotype meta-analysis) [35,36].

The overreaching conclusion after the first round of GWAS reports is that genetic heterogeneity, epistasis and complex interactions, plus demographic and environmental factors, underpin the susceptibility to ADs [13-15]. It is unclear how many genetic variants are associated with ADs, and what the immunomolecular mechanisms underlying epistasis among them are. However, a full inventory of variants is not far away and new approaches to examine epistasis will tell us how genes interact to confer either susceptibility or protection against ADs [37]. On top of this genetic view, newly published and publicly available data (for example, exome sequencing project, HapMap and the 1000 genomes project) are at par with technological approaches probing other omic layers like gene expression (for example, RNA-seq, Ribo-seq), methylation (for example, Methyl-seq; BS-seq, Bisulfite Sequencing), other epigenetic marks (for example, ChIP-seq, Chromatin Immunoprecipitation sequencing; FAIRE-seq, formaldehyde-assisted isolation of regulatory elements–sequencing) and genome structure (for example, Immuno-seq; PhIT-Seq, phenotypic interrogation via tag sequencing) [38] are gaining further attention and application to be compared and matched between their omic counterparts. Current ongoing approaches mapping genetic variation contributing to transcriptional variation, referred to as expression quantitative trait locus analyses [39,40], are assessing the role of genetic variants on the expression of genes in their vicinity; empirically, these approaches have been demonstrated to be well-powered to detect regulatory effects [41,42]. This type of post-omic information will add to current knowledge and provide new insights for mechanism and molecular processes for specific phenotyped cells and traits related to the autoimmunity phenomena.

### Pitfalls and challenges of complex trait analysis

In recent years, a plethora of new susceptibility genetic variants for ADs has emerged. The advent and advance of microarray and next-generation sequencing technologies has resulted in commercially available tools to provide and obtain genotypes and sequencing information in a fast but costly manner. This exponential production of data is reflected in the number of manuscripts reporting associations of hundreds of loci to ADs. Thus far, the human leukocyte antigen locus has disclosed the strongest association with ADs [43]. In the case of systemic lupus erythematosus, a simple search in PubMed reported more than 5,000 papers on the genetics of the disease. These describe more than 40 loci, replicated by several independent studies, that modify the risk to acquire the disease. However, these systemic lupus erythematosus-associated loci explain a minimal portion of the additive heritability, challenging the idea that this new genetic knowledge might allow for a better predictive and preventive assessment of ADs (that is, missing heritability). Table 1 summarizes the main pitfalls and challenges of complex trait analyses, which we will comment upon next.

**Table 1:** Pitfalls and challenges of complex trait analysis

**Pitfall and challenge**	**Perspective**
Complex epistatic interactions	- Better algorithms and control for phenotype and subphenotype studies. Data analysis is the next most expensive tool to develop.
Genetic heterogeneity	- Larger size cohorts.
Pleiotropy	- Familial studies to control for environmental and stochastic factors.
History of mutations and difference in allele frequencies.	- Description and study of population genetic structure in light of reported information from other reported and publicly available data.
Population stratification	- Usage of newly reported algorithms for admixture analysis and pan-meta-analysis approaches.
Genetics in admixed populations	
Statistical power and sample size	- Correspondence in the use of specific clinical criteria or diagnostic biomarkers to define phenotypes to enhance prediction and diagnosis.
Refining the phenotype - subphenotypes	Development and application of bioinformatical approaches to classify disease as quantitative and categorical entities.
Family based studies versus case–control studies	Application of classical genetic and epidemiological tools to characterize new information available for other ‘omic’ layers in the context of the genome from a familial and population viewpoint.
Gene-environment interaction	Further research in environmental factors that might influence onset of disease (for example, tobacco, coffee consumption, organic solvents)
Post-genomic era (‘omics’)	Use of the publicly available ‘omic’ information already reported (for example, ENCODE, GEO, HapMap, 1000 genomes project) to explore, replicate and hypothesize new experimental functional designs.
Personalized medicine	Genomic medicine-generated information to be applicable from the bench to bedside and also from the bedside to bench.
Pharmacogenomics	Disentangle markers capable of predicting and diagnosing risk of disease even before onset of symptoms and signs.

Two major challenges in studying ADs are the genetic heterogeneity, referring to how a set of genetic variants might define a trait onset either by their combination or differential effect, and pleiotropy [6], where a single gene leads to multiple phenotypic expressions or disorders. As mentioned by Lehner [44], the sharp statement by Sewal Wright in the 1930s that ‘each character is affected by many characters…’ is very much true today.

Diverse human populations present different allelic and genotype structures depending upon their evolutionary and epidemiological history [45]. In addition, the effects of genotype on phenotype for any given population may depend on the environment and length of exposure to an undefined etiological insult. Differences in allele and genotype frequencies among populations reflect the contribution of evolutionary forces such as selection, genetic drift, mutation and migration [46], which might explain why some risk alleles to autoimmunity may be protective factors to infectious diseases and vice versa [47]. Immune and infectious agents have been recognized as among the strongest selective pressures for natural populations [47]. Further research regarding exploration of the interplay between infection, type of exposure, additional environmental factors (for example, microbioma) and autoimmunity will result in the discovery of multiple factors underpinning perhaps newly identified physiopathology mechanisms of ADs.

The relatively short evolutionary time since the rise of modern humans after the clash of cultures in America (500 years) is a perfect scenario to dissect specific immunity associated with infectious diseases and its role in predisposition to ADs. Classical examples are Chagas’ disease (originally found in America and absent in other continents) and typhoid fever (brought to America by the Spaniards conquers). Indeed, it is not only the knowledge that might be contributed by this type of population, but also the specific and direct epidemiological and health care approach that must be provided to them. Admixed populations such as Afro-American and Latin-American are often medically underserved and bear a disproportionately high burden of disease. Thus, given the diversity of their genomes, these populations have both advantages and disadvantages for genetic studies of complex phenotypes [48]. Advances in statistical methodologies that use genetic contributions from ancestral populations contributing to the current admixed population have proven to be a powerful method to leverage the confounder effect of ancestry, and this information is used to identify chromosomal segments linked to disease [46].

Consequently, there is a need to explore genetic associations in diverse populations. Proper matching of cases and controls is a major consideration for GWAS, as well as in any case–control association study. The use of ancestry informative markers either to match or exclude cases and controls given specific patterns of genetic stratification allows us to overcome this limitation, diminishing the possibility of reaching spurious associations as a consequence of case–control ethnic microdifferentiation.

Determinants of statistical power such as sample size, disease heterogeneity, pedigree and genotyping errors, as well as the effect of the type and density of genetic markers, are a key factor in genetic studies. Studies should either have sufficient power to detect a small effect size of multiple genes or consider the use of extreme and well-defined phenotypes to detect the effect of major genes [30,31].

The term ‘metagenomics’ defines the set of mechanisms by which a community of microorganisms interacts, lives and infects animal tissues. New metagenomic approaches have disclosed crucial information about the shaping of resistance, susceptibility and loss of auto-tolerance for both infectious and ADs [49]. Indeed, new reports demonstrate that host-gene-microbial interactions are major determinants for the development of ADs. Commensal microbial communities may alter sex hormone levels and regulate AD fate in individuals with a high genetic risk load [50].

Although ADs are often diagnosed according to classification criteria, they share similar subphenotypes including signs and symptoms, non-specific autoantibodies and high levels of cytokines, which are prone to taxonomic problems [51]. ADs have a heterogeneous spectrum, the disease course differs from patient to patient and through different phases within the same patient [52]. Refining the phenotype will make the effect of certain genes in the sample more easily detectable [4]. Genetic effects may be stronger for extremes of the risk factor distribution (for example, people with onset at a very young or very old age) and for particular presentations. Therefore, restricting the sample to patients with specific characteristics, or minimizing the effect of known environmental confounders will increase the chances for genetic research to be successful.

Disease heterogeneity should be minimized by considering subphenotypes or otherwise by adjusting for known sources of heterogeneity as a covariate. Meta-analysis and data pooling between different research groups can provide a sizeable study, but both approaches require a high level of vigilance about locus and disease heterogeneity when data come from different populations. Spurious associations are often due to population stratification, cryptic relatedness and differential bias [53].

GWAS have a high power to detect common variants of high or moderate effect. For weaker effects (for example, relative risk <1.2), the power is greatly reduced, particularly for recessive loci if the frequency of the variant is common (that is, rare variants) [54]. Larger size cohorts can be used to study common diseases, but meta-analyses and data pooling are required to attain a study size of sufficient magnitude for many other diseases [53]. GWAS approaches are known to be poor in detecting effects from rare alleles (that is, frequency <5%), but novel methods and technology, such as exome and whole genome sequencing will fill this gap to further support the genetic commonality of autoimmune traits [55]. However, once a polymorphism has been found to be associated with a trait, it’s functional relevance must be examined and its biological effect on such a trait understood (that is, functional genomics).

Recent advances in multiplexed assay technology are taking us closer toward the identification of ‘actionable markers’, capable of informing and providing biological metrics of use in clinical practice. Not only will they help gain insights into the onset, remission and exacerbation of a pathology, they will improve and enhance treatment, diagnosis and classification [56].

### What is next?

Genomics normally implies the use of sequence and genome information to annotate, describe and curate functionality and structure, in order to decipher and disentangle functionality and organization. New ‘omics’ approaches are starting to take this further by correlating and matching layers of genome-wide information to explain and to explore mechanisms of interaction between genetic and environmental factors. Significant advances in human ‘omics’ are giving rise to new possibilities in medicine, such as clinical bioinformatics [57] and translational bioinformatics [58]. All these options lead to one common premise: ways of mining meaningful information from the vast amount of ‘omics’ data being generated. In this sense, application of comprehensive molecular information to clinical settings is been referred to as ‘genomic medicine’ [59] with the ultimate goal to nurture, improve and frame personalized medicine. A genomic medicine approach will always require participation at a multidisciplinary research expertise level.

Personalized medicine is committed to survey, monitor and diagnose risks to provide patients with a specific treatment, taking into account their particular genetic profile and molecular phenotype. Thus evaluation, comparison, correlation, cross-matching and interaction of the nascent ‘omic’ information would not only aid in the prediction, diagnosis and treatment at the individual level but also provide insights into the physiopathological mechanisms of disease onset and progression. For such purposes, an integrative personal ‘omics’ profile such as the one suggested by Chen *et al*. [60] will be useful to examine as many biological components as possible. Although these components might change during healthy and diseased states, this information combined with genomic information will be useful to estimate disease risk and gain new insights into diseased states [60]. Disease would be considered as a hierarchical biological system composed of molecular and functional cell, tissue and organ interactive networks. Any aberration in one or more networks will not only have local effects but also systemic effects because no cell, tissue or organ is isolated or independent.

Last but not least, safeguarding for all study participants, whether healthy or affected, and studied family members has to be warranted. Individuals are the ‘why’ behind this overhauling of ‘omic’ and genomics approaches and research, thus their legal rights and *status quo* have to be defined in order to eventually be successful in applying genomic-based medicine for the benefit of human kind. We shall not forget the understated idea ‘…we should not only be interested in the human genome but also in the human beings that carry it’ [61].

## Abbreviations

ADs: Autoimmune diseases; GWAS: Genome-wide association study.

## Competing interests

The authors declare that they have no competing interests.

## Authors’ contributions

JMA designed the review. JC, MAB and JMA jointly wrote the manuscript. All authors read and approved the final version of the manuscript.
